# Molecular Modelling of the Emergence of Azole Resistance in *Mycosphaerella graminicola*

**DOI:** 10.1371/journal.pone.0020973

**Published:** 2011-06-27

**Authors:** Jonathan G. L. Mullins, Josie E. Parker, Hans J. Cools, Roberto C. Togawa, John A. Lucas, Bart A. Fraaije, Diane E. Kelly, Steven L. Kelly

**Affiliations:** 1 School of Medicine, Institute of Life Science, Swansea University, Swansea, United Kingdom; 2 Department of Plant Pathology and Microbiology, Rothamsted Research, Harpenden, Hertfordshire, United Kingdom; 3 Embrapa Recursos Genéticos e Biotecnologia, Laboratόrio Bioinformática, Parque Estação Biológica –Final WS norte, Brasilia, Brazil; University of Kent, United Kingdom

## Abstract

A structural rationale for recent emergence of azole (imidazole and triazole) resistance associated with CYP51 mutations in the wheat pathogen *Mycosphaerella graminicola* is presented, attained by homology modelling of the wild type protein and 13 variant proteins. The novel molecular models of *M. graminicola* CYP51 are based on multiple homologues, individually identified for each variant, rather than using a single structural scaffold, providing a robust structure-function rationale for the binding of azoles, including important fungal specific regions for which no structural information is available. The wild type binding pocket reveals specific residues in close proximity to the bound azole molecules that are subject to alteration in the variants. This implicates azole ligands as important agents exerting selection on specific regions bordering the pocket, that become the focus of genetic mutation events, leading to reduced sensitivity to that group of related compounds. Collectively, the models account for several observed functional effects of specific alterations, including loss of triadimenol sensitivity in the Y137F variant, lower sensitivity to tebuconazole of I381V variants and increased resistance to prochloraz of V136A variants. Deletion of Y459 and G460, which brings about removal of that entire section of beta turn from the vicinity of the binding pocket, confers resistance to tebuconazole and epoxiconazole, but sensitivity to prochloraz in variants carrying a combination of A379G I381V ΔY459/G460. Measurements of binding pocket volume proved useful in assessment of scope for general resistance to azoles by virtue of their accommodation without bonding interaction, particularly when combined with analysis of change in positions of key amino acids. It is possible to predict the likely binding orientation of an azole molecule in any of the variant CYPs, providing potential for an in silico screening system and reliable predictive approach to assess the probability of particular variants exhibiting resistance to particular azole fungicides.

## Introduction

*Mycosphaerella graminicola* causes Septoria leaf blotch, the primary foliar disease of winter wheat in most western European countries [1]. Control of the pathogen now relies on the application of azole fungicides which are demethylase inhibitors (DMIs) that inhibit CYP51 activity. CYP51 is a cytochrome P450 that catalyses the oxidative removal of the 14α-methyl group of lanosterol or eburicol in yeasts and fungi - an essential step in the production of sterols. Azoles bind as the sixth ligand to the haem in CYP51 via the unprotonated N atom thus occupying the active site and acting as non-competitive inhibitors [2].

Many different amino acid alterations (substitutions and deletions) have been associated with azole resistance in the MgCYP51 protein of western European *M. graminicola* populations [3], [4], [5], [6], [7]. Similarly, mutations corresponding to azole resistance have also been identified in other fungal CYP51s, including the opportunistic human pathogens *Candida albicans* [8] and *Aspergillus fumigatus* [9]. In *M. graminicola* CYP51 substitutions include Y137F which confers resistance to triadimenol [6], I381V which confers resistance to tebuconazole [4] and V136A that confers resistance to prochloraz [6]. Of particular interest is a deletion of two amino acids Y459/G460 seen in many recent populations [7], located in a fungal specific region of CYP51. This deletion and other single amino acid substitutions of residues 459–461 are frequently found in populations exhibiting increased resistance to azole compounds, and a large number of multiple alterations have arisen in *M. graminicola* CYP51 [6], [7]. Such multiple changes have previously been identified in azole resistant *Candida albicans* [10], but not on the same scale. It is likely such multiple alterations have occurred in *M. graminicola* CYP51 by point mutation, intragenic recombination [5] and selection by successive treatments over time with different azoles and azole mixtures. In addition it has been suggested that ascospores have been spread with the prevailing wind (from west to east) over Europe [7]. This has resulted in replacement of wild type isolates in current European populations by a series of more complex CYP51 variants, the most prevalent in the UK *M. graminicola* population now being L50S S188N A379G I381V ΔY459/G460 N513K [7].

Although no fungal CYP51 has been crystallised to date, homology models based on the crystal structure of soluble CYP51 from Mycobacterium tuberculosis [11] have been proposed. Prior to the elucidation of this structure, the CYP51 of *C. albicans* was modelled by molecular dynamics based on the structure of *Pseudomonas putida* P450cam [12], and this model was used to rationalise the differential inhibition with azoles [13].

In recent years, the Cytochrome P450 enzymes of a number of species have been crystallised, with over 350 structures deposited on the PDB by mid 2010, providing a basis for the first time for reliable multi-homologue modelling of wild type and mutant CYP51 structures. This should provide an insightful approach for assessing the relative structural impact of single and multiple alterations on the likely effects on azole binding in CYP51s.

Successful amino acid alterations may exert an effect in one of several ways that could lead to target protein mediated resistance to a given group of chemical compounds. Azole resistance may occur due to alterations that invoke a conformational change that removes a specific residue (or residues) away from interaction with an azole. Alternatively, a key residue may be directly substituted. Either of these can result in the azole not being accommodated normally within the active site with regard to the coordinating haem (this may include occlusion of the azole from the active site) or may lead to a lower azole binding affinity due to changes in interaction with surrounding residues. Multiple alterations, of course, may result in higher or cross-resistance to azoles through a cumulative effect or they may be compensatory, restoring or improving CYP51 function which may otherwise reduce the fitness of the variant strain. It is likely that the scope for changing the gross architecture of the haem pocket is constrained by the evolutionary requirement of the molecule to maintain its native functions.

We have modelled alterations individually and in combination and simulated the docking of four azoles, epoxiconazole, tebuconazole, triadimenol and prochloraz. We discuss our models with respect to the changes commonly seen in the field and the selection pressures that are likely to have brought about these alterations. We present a structural rationale for resistance associated with CYP51 changes in *M. graminicola*. With our modelling approach, it is possible to assess the likely binding of any azole in any of the variant proteins, providing the potential for an *in silico* screening system for azole binding in fungal CYPs. Although other mechanisms of resistance to azoles may contribute to the overall phenotype (overexpression of *CYP51* and upregulation of efflux transporters) [14], the affinity of an azole for a CYP51 variant is fundamental to its functioning as an inhibitor, and new alterations and combinations of alterations in CYP51 associated with azole resistance continue to emerge [15]. Therefore molecular modelling will provide insights into azole binding in *M. graminicola* CYP51 and will aid in the design of new inhibitors and possibly the prediction of the likely resistance phenotype of a strain. It may also be possible to predict likely alterations which may occur as a result of azole treatment in the future. Furthermore the information generated in *M. graminicola* CYP51 models may be applicable to other fungal CYP51s resistant to azoles. The results presented here provide evidence of a robust protein model which may now be used to inform and predict future resistance in the field which will be further confirmed by laboratory tests.

## Methods

### Homology Modelling

Structural modelling of wild type and 13 mutant Mycosphaerella graminicola CYP51 (MgCYP51) sequences was carried out using an automated homology modelling pipeline built with the Biskit structural bioinformatics platform [16], which scans the entire PDB for candidate homologies. This work was based on the PDB as compiled on 15 February, 2010, at which time there were 316 CYP structures available. The wild type sequence used is of strain IPO323. The genome sequence of this strain is now publically available (http://genome.jgi-psf.org/Mycgr3/Mycgr3.home.html). The naturally occurring alterations were subsequently manually incorporated into the wild type sequence. The pipeline workflow incorporates the NCBI tools platform [17], including the BLAST program [18] for similarity searching of sequence databases. T-COFFEE [19] was used for alignment of the test sequence with the template. Homology models were generated over 10 iterations of the MODELLER program [20]. All models were visualized using the molecular graphics program Chimera [21]. CYP homologues are selected by the pipeline as those with the highest identity to the test sequence. Rather than simply replacing the candidate amino acid(s) in a model of wild type MgCYP51 each sequence is modelled separately. Therefore each of the MgCYP51 variants may be modelled on a different selection of homologues (table 1) depending on the identity shared with available CYP structures resulting in a model which reflects the global structural differences that result from amino acid alterations. Root mean square deviation (RMSD) of models compared to wild type were calculated using Chimera [21] to attain a measure of global structural change, and residues within given distance ranges of the docked azoles were identified using the zone function in the same program.

**Table 1:** Modelled *M. graminicola* CYP51 mutants, homologues adopted by the automated modelling pipeline and the highest percentage homology achieved.

Variant	% Homology	PDB Homologues
Wild type (wt)	33.6	3DBG 3G1Q 2CIB 3I3K 3GW9 3L4D
L50S	34.5	3DBG 3G1Q 2CIB 3I3K 3GW9 3L4D
Y137F	33.3	3DBG 3G1Q 2CIB 3I3K 3GW9 3L4D
Y459D	34.2	3DBG 3G1Q 2CIB 3I3K 2VE3 3GW9 3L4D
G460D	33.3	3DBG 2Q9F 3G1Q 2CIB 3I3K 2VE3 3GW9 3L4D
L50S Y461H	32.7	3DBG 2Q9F 3G1Q 2CIB 3I3K 2VE3 3GW9 3L4D
L50S Y461S	32.7	3DBG 2Q9F 3G1Q 2CIB 3I3K 2VE3 3GW9 3L4D
L50S I381V Y461H	33.1	3DBG 3G1Q 2CIB 3I3K 2VE3 3GW9 3L4D
L50S V136A Y461H	33.1	3DBG 2Q9F 3G1Q 2CIB 3I3K 2VE3 3GW9 3L4D
L50S S188N N513K	34.2	3DBG 3G1Q 2CIB 3I3K 3GW9 3L4D
L50S S188N ΔY459/G460 N513K	29.1	3DBG 2CD8 2Q9F 3G1Q 2CIB 3I3K 3GW9 3L4D
L50S S188N I381V ΔY459/G460 N513K	33.1	3DBG 3G1Q 2CIB 3I3K 3GW9 3L4D
L50S V136A S188N ΔY459/G460 N513K	29.3	3DBG 2CD8 2Q9F 3G1Q 2CIB 3I3K 3GW9 3L4D
L50S S188N A379G I381V ΔY459/G460 N513K	33.6	3DBG 3G1Q 2CIB 3I3K 3GW9 3L4D

### Measurement of binding pocket volume

The volume of the haem cavity of the wild type and variant protein models was determined using Pocket-Finder written by Alasdair Laurie and Richard Jackson, University of Leeds, UK, (http://www.modelling.leeds.ac.uk/pocketfinder/) which is a pocket detection algorithm based on Ligsite [22].

### Azole docking

The azole molecules (triadimenol, tebuconazole, epoxiconazole and prochloraz) were docked by superimposition within the active site cysteine pocket, with the unprotonated N atom coordinated by the haem iron atom. The entire azole ring proximal to the haem group was precisely positioned according to the haem position and fluconazole binding in PDB structure 3L4D (deposited December 2009), the crystal structure of sterol 14α demethylase from *Leishmania infantum* (Lepesheva et al., to be published). We selected the CYP51 structure from *Leishmania infantum* (Lepesheva et al., to be published) as the template for azole docking on the basis of it possessing the highest identity with *M. graminicola* CYP51 of any of the structures co-crystallised with an azole molecule. The variant protein structures were also placed by superimposition and screened for conflicts with docked azoles at a required separation threshold of 0.6 Å. Residues within given distance ranges (<3.0 Å, 3.0–3.5 Å, 3.5–4.5 Å) of the docked azoles were identified using the surface zone function in the Chimera molecular graphics program and H-bonds were predicted [21].

### *M. graminicola* isolate azole sensitivity testing

Sensitivity assays were modified from Fraaije et al. (2007) [4]. A 100 µl aliquot of 2× Sabouraud Dextrose Liquid Medium (SDLM; Oxoid, Basingstoke, UK) amended with decreasing concentrations of epoxiconazole (75, 20, 5.3, 1.4, 0.38, 0.101, 0.027, 0.007, 0.002, 5.00E-04 and 1.36E-04 mg l^−1^), tebuconazole (75, 27, 9.9, 3.6, 1.3, 0.48, 0.17, 0.063, 0.023, 0.008 and 0.003 mg l^−1^), triadimenol (75, 25, 8.3, 2.8, 0.93, 0.31, 0.10, 0.034, 0.011 and 0.004 mg l^−1^) and prochloraz (15, 3.0, 0.60, 0.12, 0.024, 0.005, 9.60E-04, 1.92E-04, 3.84E-05, 7.68E-06 and 1.54E-06 mg l^−1^) was added to wells of flat-bottomed microtitre plates (TPP 92696 test plates, Trasadingen, Switzerland). After 7 days growth at 15°C on YPD to ensure yeast-like growth, isolates were suspended in 5 ml of sterile distilled water. Aliquots of 100 µl of isolate spore suspensions (2.5×10^4^ spores ml^−1^) were added to each well. Plates were incubated for 3 days at 23°C, and growth measured by absorbance at 630 nm using a FLUOstar OPTIMA microplate reader (BMG Labtech GmbH, Offenberg, Germany). Fungicide sensitivities were determined as 50% effective concentration (EC50) using a dose response relationship according to the BMG Labtech Optima Software. Resistance factor (RF) of each isolate was calculated as fold change in EC50 compared to wild-type isolates.

## Results and Discussion

Wild type MgCYP51 and a selection of MgCYP51 variants were modelled. Amino acid alterations currently observed in the field were modelled in order to elucidate the contribution of single and multiple alterations on the resistance and activity profile of MgCYP51. We used the PocketFinder (http://www.modelling.leeds.ac.uk/pocketfinder/) algorithm to calculate the predicted size of the pocket volumes of our 14 models and the root mean squared deviation (RMSD) as a measure of the overall change in the conformation of our models compared to the wild type model. Together these give a measurement of the effect an alteration or combination of alterations is likely to cause on the structure of MgCYP51 (table 2). In addition, we have used molecular modelling to simulate the docking of epoxiconazole, prochloraz, tebuconazole and triadimenol in wild type and mutant models to gain an insight into the mechanism behind the associated azole resistance. We have considered the effect that individual alterations may contribute and the overall profiles expected in variants carrying multiple changes currently observed in the field.

**Table 2:** Binding pocket volumes of modelled *M. graminicola* CYP51 mutants, and their RMSD values compared to wild type, ordered according to cavity volume.

Variant	Volume(Å^3^)	RMSD	No. residues within 3 Å of azole	No. polar residues within 4.5 Å of azole
			E P T Tri	E P T Tri
**L50S I381V Y461H**	1217	0.954	7 6 6 6	4 4 4 4
**L50S Y461S**	1460	1.018	5 6 5 3	3 2 4 2
**wild type (wt)**	1685	---	6 5 6 4	3 3 4 4
**L50S V136A Y461H**	1971	1.044	8 6 6 8	5 4 5 5
**Y459D**	2263	0.962	6 5 4 4	1 3 3 3
**L50S**	2269	1.076	4 4 4 4	4 3 6 4
**L50S Y461H**	3451	0.971	5 4 3 4	4 5 5 3
**G460D**	3501	0.958	6 6 7 5	2 3 3 3
**L50S S188N N513K**	3754	0.905	2 2 3 2	2 3 3 1
**Y137F**	3769	0.798	3 3 4 2	2 3 3 3
**L50S V136A S188N ΔY459/G460 N513K**	4355	1.051	3 4 4 1	0 1 1 1
**L50S S188N A379G I381V ΔY459/G460 N513K**	5060	0.912	4 3 2 2	1 0 2 1
**L50S S188N I381V ΔY459/G460 N513K**	5204	0.998	4 4 3 1	1 1 1 1
**L50S S188N ΔY459/G460 N513K**	5725	0.983	3 3 3 2	1 2 3 1

Wild type shaded, variants that occur in the field shown in bold.

### Wild type *M. graminicola* CYP51

**Figure 1:**
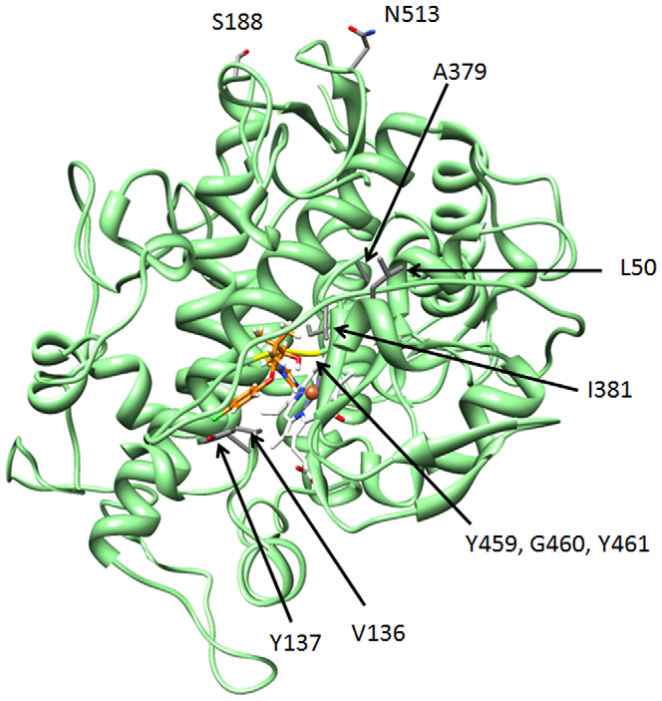
Wild type CYP51. Wild type CYP51 binding triadimenol, showing the location in the wild type protein structure of the residues subject to alteration; I381 and Y137 labelled, V136, in grey, neighbouring Y137, triadimenol in orange and the Y459/G460 region in yellow. The chloride group (green) of triadimenol is predicted to form a weak hydrogen bond with Y137. The I helix of the protein is shown running down the left hand side of the bound azole and the K helix behind I381 extending down behind the haem group. Figure 1 shows the location in the wild type protein structure of the residues subject to alteration. L50 and A379 do not directly border the binding pocket, but form part of secondary structures that are immediately adjacent to the cavity. S188 and N513 are located on the outside of the protein, far removed from the binding regions.

*M. graminicola* CYP51 is predicted to possess a typical P450 fold with the generally conserved structural core formed by helices E, I, J, K, and L around the haem prosthetic group (figure 1), similar to the recently characterised human CYP51 [23], and the access to the haem cavity guarded by the loop between helices B and C. The predicted volume of the wild type protein is 1685 Å^3^, including the space occupied by the haem. A total of 86 residues were identified as lining the pocket of the wild type protein (text S1), including 5 residues (V136 (on the B–C loop), I381 (K–L loop), Y459, G460 and Y461) observed as sites of alteration leading to azole resistance in *M. graminicola* [24]. Figure 1 shows the location in the wild type protein structure of the residues subject to alteration. V136 and Y137 reside on the access channel end of the binding pocket, while I381, Y459, G460 and Y461 are at the haem end. L50 and A379 do not directly border the binding pocket, but form part of secondary structures that are immediately adjacent to the cavity. S188 and N513 are highly exposed on the outside of the protein, far removed from the binding regions.

The numbers of residues bordering the binding pocket and predicted to be within 3 Å of epoxiconazole, prochloraz, tebuconazole and triadimenol are shown in table 2 (the residues predicted to be within 3.0–3.5 Å and 3.5–4.5 Å of docked azoles are listed in table S1). The docking of azoles to the wild type MgCYP51 reveals a pattern of residues immediately proximal to the azole docking site known to be subject to mutation which results in azole resistance (V136, Y137, I381, Y459, G460, and Y461). All these residues are implicated, for instance, by the interactions of triadimenol docking (closer than 4.5 Å). By this analysis, L50, S188, A379 and N513 are confirmed to be located away from the binding region. This modelling is consistent with the use of azoles leading to the emergence of the key resistance mutations at Y137, I381V and Y459.

### Impact of alterations

The effect of individual alterations upon CYP51 structure was assessed by modelling the altered forms of the protein. Test sequences were modified accordingly and input into the modelling pipeline. Table 1 details the changes in homologues adopted by the automated modelling pipeline and changes in sequence homology, which lead to the structural changes observed in the modelled structures. The proximity of residues to each of the azoles for which binding was simulated was assessed and compared with the wild type protein.

For the purpose of discussion of the impacts of alterations, the variants are divided into three groups; i) Alterations that do not affect azole binding, ii) Alterations affecting the size and accessibility of the binding pocket, iii) Alterations affecting the localisation of residues interacting with azoles.

### i) Alterations that do not affect azole binding

#### L50S

The inclusion of L50S on its own causes only a slight increase in cavity volume (table 2). The L50S substitution appears in the majority of subsequent combinations of multiple mutations and its location away from the cysteine pocket (L/S50 is not within 4.5 Å of any of the docked azoles in any of our 14 models (table S1) and its marginal impact on protein conformation indicate that it may indeed represent a natural variant in its own right which is not associated with azole resistance. The L50S alteration also does not affect activity as shown by expression in yeast [25].

#### L50S S188N N513K

L50S S188N N513K is a natural variant found in wild type populations in New Zealand sensitive to all azoles (Cools et al., unpublished). Although the model shows a significant increase in the volume of the haem cavity (table 2), and generally fewer residues in close proximity to the azoles, key residues for interaction, namely Y137 and I381 are as close or closer to the docked azoles than in the wild type protein (table S1), being within 3.5 Å of all azoles, an arrangement consistent with general azole sensitivity. V136 is within 4.5 Å of triadimenol, tebuconazole and epoxiconazole, but not prochloraz, also like the wild type protein.

Leroux et al., (2007) have suggested that the mutations recorded at positions 50, 188, 379 and 513 do not seem to be related to DMI resistance, but suggest that *M. graminicola* populations might be constituted of two independent entities that represent sympatric species [6]. They suggest that S188N and N513K (along with L50S) are always seen together and may have originated from the same haplotype rather than having been selected through conferring azole resistance [6]. Brunner et al [5] have proposed an alternative hypothesis, that rapid evolution of the CYP51 gene has led to two distinct populations of molecules, one based on the accumulation of a series of point mutations within the ancestral wild-type template and another that is highly diverged as a result of a few intragenic recombination events that brought together the different point mutations from different regions of the gene.

This genotype may exhibit general sensitivity to the above azoles and might present an increased selective pressure for mutation at the 136 and 381 positions. This is supported by the observation that the L50S variant model brings I381 into closer interaction with triadimenol (from within 4.5 to <3.0 Å), tebuconazole and epoxiconazole (now within 4.5 Å) and remains within 3.0 Å of prochloraz (table S1). There is a reduction in the overall number of residues within 4.5 Å of triadimenol (13 to 9) and prochloraz (11 to 10), but an increase in those within 4.5 Å of tebuconazole (12 to 14). There are also more polar residues within 4.5 Å of tebuconazole and epoxiconazole (table 2).

### ii) Alterations affecting the size and accessibility of the binding pocket

The predicted structure of the L50S I381V Y461H variant displays a notable constriction of the binding pocket, while the cavity of L50S S188N N513K is more than double the size of the wild type pocket, suggesting that the slight increase in cavity volume brought about by L50S on its own is modified considerably by the other alterations with which it is combined.

### Variants including I381V

Several of the multiple alterations modelled are seen in current populations. The structural models will be discussed in relation to the resistance profiles observed and compared to wild type structure to account for resistance, beginning with those that include the influential I381V alteration.

Multiple alterations including I381V display some sensitivity to prochloraz. L50S I381V Y461H is a common natural variant, which causes resistance to epoxiconazole and slightly decreased sensitivity to prochloraz (table 3). The predicted cavity volume of this mutant protein model is notably smaller than wild type at 1217 Å^3^ and therefore the number of residues within interaction range of docked azoles is increased (table 2).

**Table 3:** EC50 and resistance factor values of CYP51 variants and structural changes conferring resistance.

CYP51 variant	Number of strains	Epoxiconazole		Prochloraz		Tebuconazole		Triadimenol		Structural mechanism of resistance
		Mean EC50^a^(mg l^−1^)	RF^b^	Mean EC50(mg l^−1^)	RF	Mean EC50(mg l^−1^)	RF	Mean EC50(mg l^−1^)	RF	
Wild-type	4	0.003±0.001	**-**	0.016±0.005	**-**	0.072±0.026	**-**	0.864±0.227	**-**	No resistance
Y137F	4	0.015±0.004	**5.1**	0.084±0.019	**5.1**	0.262±0.042	**3.6**	14.73±3.224	**17**	Obstruction of binding site by F137, particularly triadimenol
L50S, Y461H	2	0.048±0.016	**17**	0.070±0.007	**4.3**	1.185±0.075	**16**	ND^c^		Azole interaction lost at H461
L50S, I381V, Y461H	17	0.249±0.029	**87**	0.079±0.025	**4.8**	3.882±0.327	**54**	“		Constriction of binding cavity, azole interaction lost at H461
L50S, V136A, Y461H	8	0.205±0.030	**71**	0.455±0.076	**28**	0.385±0.188	**5.4**	“		Loss of interaction between Y137 and prochloraz
L50S, S188N, ΔY459/G460, N513K	4	0.088±0.032	**31**	0.069±0.021	**4.2**	2.214±0.939	**31**	“		Massive increase in binding cavity volume. I381 near prochloraz
L50S, S188N, I381V, ΔY459/G460, N513K	4	0.196±0.072	**68**	0.078±0.025	**4.8**	2.930±0.556	**41**	“		Massive increase in binding cavity volume. V381 near prochloraz
L50S, V136A, S188N, ΔY459/G460, N513K	3	0.255±0.024	**89**	0.359±0.122	**22**	0.114±0.025	**1.6**	“		Large increase in binding cavity volume. K148 near tebuconazole.
L50S, S188N, A379G, I381V, ΔY459/G460, N513K	16	0.447±0.079	**155**	0.012±0.003	**0.7**	9.439±1.382	**131**	“		Massive increase in binding cavity volume. V381 near prochloraz

a Mean EC50 of strains with ± standard error.

b Resistance factors (RF) of strains calculated as the fold changes in EC50 compared to the mean EC50 of wild-type strains.

c not determined.

It is the incorporation of I381V that causes the decrease in the cavity volume. The single I381V substitution reduces the cavity volume to 1438 Å^3^, and similarly causes a relative decrease in the cavity volume in other mutants where it appears (table 2). Interestingly, L50S I381V and I381V have never been observed in populations in isolation, and do not complement CYP51 function in a heterologous system [25], indicating that the conformational change also results in decreased activity. Leroux and colleagues have observed high resistance of this laboratory variant to triadimenol, tebuconazole and epoxiconazole [6].

I381V is usually found in populations with changes in the 459–461 region [6], [7], indicating that CYP51 function is lost with I381V and this mutation is only selected after other alterations have occurred. Indeed, it has been proposed that a recombination event brought these two mutations together [5]. It has also been shown in the heterologous system that the combination of I381V and Y459–Y461 rescues the lethality of I381V alone [25].

I381/V381 is within 4.5 Å of docked azoles in 36 of our 56 docking simulation models (table S2) and so is clearly under substantial evolutionary pressure. The I381V substitution results in the entire 459–461 section and Y137 being worked closer to the docked azole (table 2, table S1) – substantial structural changes which may account for the lack of CYP51 activity seen and a consequent lack of selective pressure without the presence of other mutations.

L50S I381V Y461H is a common variant, resistant to tebuconazole and sensitive to prochloraz. No strict pattern of individual residues within interaction range of docked azoles can account for resistance, in fact Y137 is brought to within 3 Å of all azoles including prochloraz and the proximity of V136 with triadimenol, tebuconazole and epoxiconazole is maintained. It may be that the substitution of tyrosine by histidine at position 461 in itself may cause azole resistance, though this alone does not explain why L50S I381V Y461H causes further decreases in sensitivity to tebuconazole and epoxiconazole compared to L50S Y461H. Proximity to V381, G460 and S315 also appears to be related to observed resistance. All are out of range of tebuconazole, while V381 is within 3 Å and G460 is within 3.5 Å of prochloraz. This fits in with the observed EC50 and resistance factor values (table 3), which are markedly higher for tebuconazole than for prochloraz.

Therefore, here it appears that it is the general constriction of the cavity combined with removal of structure at the 460–461 end that may be responsible for the observed resistance to tebuconazole and epoxiconazole, while prochloraz gives a different profile, lying closely to V381 and G460. This protein also conferred impaired growth in a heterologous system [25], indicating that the conformational change also results in decreased activity.

Our modelling suggests that the main effect of the I381V alteration appears to have been to reduce the interactions of triadimenol, tebuconazole and epoxiconazole by constriction of the haem cavity at the K and L helix end, and subsequent removal from interaction range of key tyrosine and polar residues elsewhere around the cavity, rather than removal of the 381 position itself. In the wild type model, I381 is within 3 Å of prochloraz and within 4.5 Å of triadimenol, but out of range of epoxiconazole and tebuconazole. In the L50S model, I381 is generally around 1.5 Å closer to the docked azoles, being within 3 Å of prochloraz and triadimenol and within 4.5 Å of epoxiconazole and tebuconazole (table S1). I381V has not been observed in the field without L50S. Even following multiple alterations including I381V, the 381 position remains in close range of prochloraz, though not the other azoles, which appears to be the strongest factor in the retention of sensitivity to prochloraz.

### The ΔY459/G460 deletion

Alterations of residues 459 and 460 of MgCYP51 occur frequently. Docking of azoles in our models reveals these residues are within 4.5 Å in 34 of the 40 docking models that do not include the ΔY459/G460 deletion, indicating that these amino acids are important in azole binding. The residues 459–461 lie within a region (438–463) which is specific to fungal CYP51s. It is this region which renders fungal models of MgCYP51 that use only a single homologue as a template unreliable. Cañas-Gutiérrez et al. used the *Myocbacterium tuberculosis* CYP51 structure as a template for an *M. fijiensis* model and found the area between position 442–472 (their alignment) resulted in an energetic difference between the model and the scaffold [26]. In contrast, our method is not constrained to a single homologue and our results below show how important this region is in relation to azole resistance, particularly in the ΔY459/G460 mutant. Indeed, this is the first fungal CYP51 model that provides structural evidence for the importance of Y459–Y461 for azole binding.

**Figure 2:**
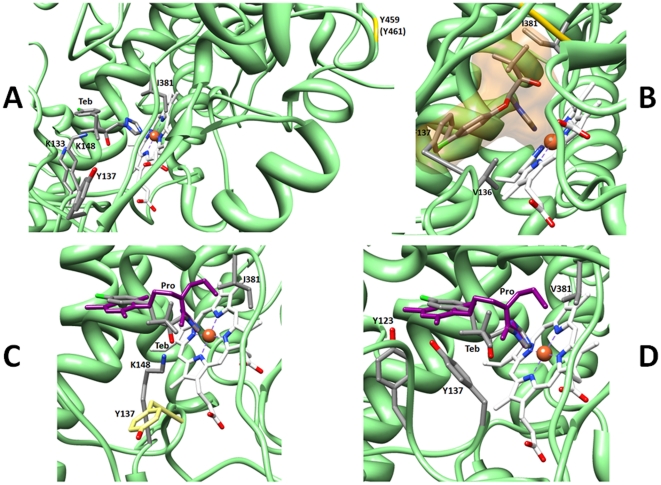
Azole docking in mutant proteins. (A) The effect of the Y459/G460 deletion, shown with tebuconazole docked. Y137 is out of range of interaction, while K133 and K148 are introduced to the pocket and are in close range of the azole. (B) Simulated docking of triadimenol in the Y137F mutant protein, showing the spatial conflicts with F137 and to a lesser extent, I381, suggesting that triadimenol binding would be significantly impaired. Triadimenol is shown in stick representation, surrounded by the external atomic surface in light orange. V136, F137 and I381 are labelled, and the 459–460 region in yellow. The reactive chloride group (green) of triadimenol is in spatial conflict with F137. (C) Resistance to prochloraz and sensitivity to tebuconazole of the L50S V136A S188N ΔY459/G460 N513K variant, showing tebuconazole (by element) and prochloraz (purple) superimposed, K148 (by element, below) out of range of prochloraz but within range of tebuconazole and Y137 (below) removed from interaction range. (D) Figure 2D. Resistance to tebuconazole and sensitivity to prochloraz of the L50S S188N A379G I381V ΔY459/G460 N513K variant, showing tebuconazole (by element) and prochloraz (purple) superimposed in docked position, V381 (grey) out of range of tebuconazole but within range of prochloraz and the hydroxyl groups of Y123 and Y137 (left) again closer to prochloraz.

The incorporation of the Y459/G460 deletion is consistently associated with large increases in cavity volume. A deletion at Y459/G460 is predicted to increase the cavity volume to 4755 Å^3^ and in combination with other mutations also increases the cavity volume (L50S S188N ΔY459/G460 N513K (5725 Å3) compared to L50S S188N N513K (3754 Å3). This increase in volume is to be expected, as the deletion results in the removal of that whole section of beta turn from the vicinity of the haem pocket (figure 2A). The removal of this region out of the haem cavity to the outside of the protein is a feature of all mutants carrying the ΔY459/G460. Variants carrying the deletion also display some of the highest RMSD values compared to wild type.

ΔY459/G460 consequently brings about a dramatic effect on the proximity of key residues to each of the azoles docked. Y137, Y461, and of course the deleted Y459 and G460, are entirely removed from the binding pocket whilst the lysine residues K133, K148 and K149 move into the pocket (figure 2A). These changes are brought about by gross rearrangement of the two sections involved, namely that containing K133, Y137, K148 and K149 and the beta turn section 450–470 containing residues 459–461 (figure 2A). In *B. graminis f. sp. hordei* the combination of the Y136F and K147Q substitution displays additive effects towards triadimenol resistance [27].

The loss of interaction with Y137 and Y459 balanced by the introduction of the two lysine residues within range for interaction is in good keeping with the observation of medium level resistance to all the azoles tested [6]. Interestingly this alteration has been predicted to have arisen in two individual events by Brunner et al. [5], further demonstrating the importance of this deletion in the development of higher level azole resistance. This is the first model to show the considerable effect of deletion of 459/460.

### L50S S188N ΔY459/G460 N513K and L50S S188N I381V ΔY459/G460 N513K

The largest change in pocket volume was found in the natural variant L50S S188N ΔY459/G460 N513K (5724 Å^3^). All the mutated residues are withdrawn from the azole binding region, except for I381 which remains within 3.5 Å and 3 Å of triadimenol and prochloraz respectively. Q313 is brought within range of tebuconazole, and H314 within reach of all the azoles. Y123 is within 3 Å of triadimenol, tebuconazole and epoxiconazole and within 4.5 Å of prochloraz. This variant demonstrates substantial resistance to epoxiconazole and tebuconazole and more modest resistance to prochloraz (table 3), which suggests that proximity to I381 is a factor in determining the extent of resistance to a given azole, but particularly prochloraz.

For the natural variant, L50S S188N I381V ΔY459/G460 N513K, the model showed the haem cavity volume increased to 5204 Å^3^ and other than V381 (within 3.5 Å of triadimenol and prochloraz), all mutated residues are removed from the binding pocket. However Y123 is within 3 Å of all azoles - this is the only polar residue found in interaction range of all docked azoles. Isolates carrying this variant are resistant to tebuconazole and sensitive to prochloraz [4], our models suggesting again that the proximity of the 381 position to prochloraz is the determining factor in the differential sensitivities observed.

L50S S188N ΔY459/G460 N513K and L50S S188N I381V ΔY459/G460 N513K may reduce sensitivity to some azoles in a similar way since Y137 and lysine residues (K133, K148 and K149) are all more than 4.5 Å from docked azoles.

### iii) Alterations affecting the localisation of residues interacting with azoles

#### Y137F

The Y137F substitution brings about a substantial increase in the size of the pocket to over twice that of the wild type (3769 Å^3^, table 2). This is the largest change in cavity volume brought about by any of the single alterations modelled. However, Y137F does not exert its effect by reducing the number of polar residues in proximity to the bound azoles (table 2) and it also does not remove the altered residue from the pocket. In the model F137 is closer to the docked azoles than Y137 (within 4.5 Å of all azoles and within <3 Å of triadimenol, table S1) and is pushed into an obstructive position prohibiting the binding of triadimenol (figure 2B). The docking of the other azoles within the haem cavity is not constrained by the occlusion of the pocket around the 137 position; rather it is the loss of the hydroxyl group incurred by the substitution of tyrosine with phenylalanine which may result in the minor decrease in sensitivity to tebuconazole, epoxiconazole and prochloraz observed. The conformational change caused by the substitution also serves to take Y459 away (around 2 Å) from the azole binding position. Y459 is within 3 Å of all docked azoles in the wild type, but in the mutant is only within 4.5 Å of triadimenol and is moved out of interaction range (>4.5 Å) of tebuconazole, epoxiconazole and prochloraz (table S1).

These modelling observations are consistent with resistance recorded in *M. graminicola* and other fungi with mutations of equivalent residues. The mutations Y132H in *Candida albicans* [28], Y136F in *M. fijiensis* [26], Y136F in *Erysiphe gramainis f.sp. hordei* [29] and Y136F in *Uncinula necator* [30] and have all been associated with azole resistance. Y137F in *M. graminicola* is specifically associated with triadimenol resistance [6]. Strains carrying the Y137F substitution are believed to have emerged due to triadimenol usage [24] and although the mutation was common in the early 1990s it is now rare in most European populations [7], having been replaced by mutations in the 459–461 region. In addition the large conformational change predicted by our model is further supported by the fact that the activity of MgCYP51 Y137F is greatly reduced, to around 10% of that of the wild type [31].

### Y459D, G460D, Y461H and Y461S alterations

Although single substitutions at positions 459, 460 and 461 all lead to an increase in cavity volume to varying extents (table 2), indeed G460D leads to a more than doubling of the cavity volume (to 3501 Å^3^), the main effects of these single substitutions are with regard to the localisation of azole-interacting residues.

With the addition of the substitution Y459C in L50S Y459C, a rare variant, Y137 is withdrawn from the pocket (>4.5 Å from all docked azoles) while I381 and Y461 remain close to the azole. Y461 is predicted to be brought closer (<3.0 Å) than in L50S or the wild type. By substitution of Y459 to cysteine, the azole proximity at that position widens when triadimaenol and tebuconazole are tested (<3 to <3.5 Å and <3 to <4.5 Å respectively).

Our modelling of the Y459D substitution shows that V136 and Y137 are withdrawn from the azole binding pocket (>4.5 Å from all docked azoles) whilst I381 is brought closer to bound triadimenol (<3 Å) and tebuconazole (<4.5 Å).

In contrast the G460D substitution shows that azoles maintain close contact with both Y137 and Y459, however, V136 is removed from interaction range (>4.5 Å). The mutated D460 residue itself is pushed into close proximity with triadimenol, tebuconazole and epoxiconazole (<3 Å).

With the Y461H substitution, the most notable difference is at the site of the mutated residue where the azole interaction with Y461 is lost due to its change to histidine. Close proximity is maintained between triadimenol and I381, indeed markedly closer proximity (<3.0 Å compared to <4.5 Å) than for the wild type (table S1). The inclusion of Y461H in L50S Y461H results in residue Y123 being closer to all the docked azoles (<3 Å) whilst Y137 and Y459 also remain close.

L50S Y461S unsurprisingly provides a similar profile to L50S Y461H - since serine has a smaller side chain than tyrosine, S461 is further from the tested azoles than Y461 >4.5 Å for all docked azoles). However, unlike Y461H, Y137 is withdrawn from the azole binding region (>4.5 Å from all docked azoles).

In summary, substitutions at positions 459–461 cause azole resistance by moving residues V136 and or Y137 further from the docked azoles, whereas the biggest impact on azole binding of alteration at Y461 is likely to be due to the loss of the tyrosine residue. An inference from our structural modelling is that the agricultural azoles impose uniformly intense selective pressure at residues between 459–461 forcing the organism toward the adoption of an evolutionary strategy of more drastic deletion rather than single substitutions in that region. This idea is supported by the fact that ΔY459/G460 is present in the most prevalent genotypes recently observed.

### L50S V136A Y461H

V136A has not been found alone in *M. graminicola*. Similar to I381V, when introduced alone and expressed in yeast it is lethal and can be partially rescued by combining with Y459–Y461. However, L50S V136A Y461H is a quite common variant that causes resistance to prochloraz and it has been postulated that the V136A substitution has been selected by the use of this imidazole [4]. Although V136 is not within 4.5 Å of prochloraz in any of our mutants, our model agrees with this finding. Rather than prochloraz resistance being directly via a change in the interacting residue it is proposed that a conformational change results in Y137 and Y123 moving further from docked prochloraz that reduces affinity.

The haem cavity volume of the natural variant, L50S V136A Y461H, is only slightly increased to 1971 Å^3^ but more residues are within 3 Å of docked azoles than in the wild type. In particular, Y137 and residue 461 are closer to all docked azoles, though for prochloraz, Y137 is more than 4 Å away. However, V136 proximity with triadimenol, tebuconazole and epoxiconazole is lost. This variant causes resistance to prochloraz, far greater than with Y461H alone. Compared to L50S Y461H, the main difference between the two model structures is that I381 is about 1 Å further away in L50S V136A Y461H.

V136 is calculated as being closer than 4.5 Å to the docked azole in 15 of our 56 azole docking models, but not for prochloraz binding (table S1). In L50S V136A Y461H, residues 459–461 are closer to the docked azoles than in L50S Y461H.

With L50S V136A S188N ΔY459/G460 N513K, the haem cavity volume is increased to 4355 Å^3^ causing a great reduction in the number of residues within 3 Å of docked azoles (table 2). K148 is within 3 Å of tebuconazole, but further away (3.5–4.5 Å) from the other azoles, which correlates with the observed sensitivity to tebuconazole and conversely the observed resistance to epoxiconazole and prochloraz (table 3, figure 2C).

### Inclusion of A379G

The inclusion of A379G in L50S S188N A379G I381V ΔY459/G460 N513K decreases the cavity volume only slightly to 5060 Å^3^ from 5204 Å^3^ (compared to L50S S188N I381V ΔY459/G460 N513K, table 2). In this variant, Y137 is around 4 Å from epoxiconazole and tebuconazole (figure 2D), which gives some scope for resistance with only the weakest form of H-bond being possible. Indeed, both V136 and Y137 are within interaction range of triadimenol, tebuconazole (figure 2D) and epoxiconazole, but the conspicuous proximity of V381 to prochloraz alone is repeated. V381 is around 2 Å closer to prochloraz than the other azoles (figure 2D). Stammler et al., (2008) [7] have found that this construct is highly resistant against epoxiconazole and tebuconazole, but sensitive to prochloraz (table 3). It is interesting that the incorporation of the A379G alteration has little impact upon the binding capacity of the nearby 381 position (table S1), which can be associated with interaction with prochloraz and consequent sensitivity (table 3). The more compact structure of the valine side chain might allow richer hydrophobic interactions with prochloraz than the wild type isoleucine side chain accounting for the sensitivity seen to prochloraz in this strain.

The presence of I381V in combination with other alterations increases following the introduction of tebuconazole and decreases after prochloraz, with no difference following epoxiconazole [4]. This sensitivity to prochloraz correlates with proximity to Y123 and particularly, V381. These findings support a notable role for Y123 and V381 in maintaining sensitivity to prochloraz, and suggest that despite their proximity, K133 and V136 are not important in the interactions with triadimenol, tebuconazole and epoxiconazole.

### Recent strains and compensatory sensitivity for tebuconazole and prochloraz

In the recent isolates, it is interesting to note that the I381V and A379G alterations increase resistance factors to tebuconazole but lower them for the imidazole prochloraz, whereas the V136A alteration increased resistance to prochloraz [4], [6], [7]. It appears therefore that recent alterations serve to convey resistance to one of either tebuconazole or prochloraz, which is compensated by sensitivity to the other. This suggests that continued administration of combinations of azoles is feasible, providing that alterations are restricted to combinations of the currently altered residue set.

The final column of table 3 provides a summary of the main structural mechanisms underlying the resistance profiles observed for the main resistance variants. Three distinct mechanisms emerge, namely obstruction of binding or loss of interaction due to the replacement of a specific residue; constriction of the binding cavity by incorporation of the I381V alteration, resulting in the cavity being inaccessible to larger azoles such as tebuconazole; extensive increase in the volume of the binding cavity, leading to isolation of the azole from the key interacting residues.

### Alterations at other sites

A consistent feature of all the models of azole docking in this study is the proximity of A311. It is the only residue predicted to be closer than 4.5 Å to epoxiconazole, triadimenol, tebuconazole or prochloraz. The equivalent residue has previously been identified in *M. fijiensis* as being mutated (A313G) though a link to azole resistance could not be confirmed [26]. An alignment of *M. graminicola* and *M. fijiensis* CYP51 sequences with *Mycobacterium tuberculosis* reveals that the residue lies in the substrate signal recognition sequence 4 (SRS4) and is also located within the I helix [26]. Substitutions at this residue (A311G) have also been identified in *M. graminicola*, and indeed used to be more common (Fraaije et al., unpublished). A mutation affecting A311 is likely to further reduce DMI sensitivity. The modelling also implicates the neighbouring residue G312, though to a lesser extent (table S2), and the G312A substitution has also been found previously. D134 is a residue in close proximity to bound triadimenol and epoxiconazole. Interestingly the D134G substitution used to be quite rare but now appears on the increase (Cools et al., unpublished). This illustrates the capacity of the modelling approach to not only rationalise the effects of alterations that have been characterised in the laboratory but also to identify other residues that may susceptible to alteration in the past or in the future and to test theoretical combinations in a predictive manner.

### Conclusion

We have modelled the *Mycosphaerella graminicola* CYP51 enzyme along with 13 altered sequences containing one or a combination of amino acid alterations which have been associated with azole resistance. The multi-homologue models were generated in a wholly automated fashion, blind of prior functional inference and unbiased by homologue selection. The structural models support observations of resistance in field populations indicating that we have created a robust model which may be used for prediction of resistance to azoles and possible new mutations that may arise.

Our molecular modelling provides a structural rationale for the emergence of azole resistance in *M. graminicola* and suggests an element of compensation between atomic rearrangement and cavity extension in the later variants has occurred in the evolutionary response of MgCYP51 to different fungicide exposures. Extending the volume of the haem cavity while limiting structural rearrangement would appear to be a feasible evolutionary solution that allows the accommodation of the larger azole molecules without their engagement in interaction, while limiting rearrangement of specific side chains important for the general function of the enzyme that is essential for growth.

Our molecular modelling of *M. graminicola* CYP51 and its variants provides a robust structure-function rationale for the binding of azoles including the importance of Y137 in the binding interactions and valuable insights of mechanisms conferring resistance. Examination of the wild type binding pocket implicates several residues in close proximity to the positions of the bound azole molecules that are subject to alteration, presenting a picture of the various ligands being spatially constrained by specific regions bordering the pocket that become the focus of genetic mutation events and those following mutations result in subsequent resistance to that group of related compounds. The most striking of these is the previously structurally uncharacterised 459–461 region, which is the focus of several different single site substitutions and a 2 amino acid deletion, at 459–460 in various resistant strains. In structural terms, the deletion is strikingly effective, resulting in the removal of the entire beta turn section from the vicinity of the binding pocket. The models account for several observed functional effects of specific mutations, including the loss of triadimenol sensitivity in isolates possessing the Y137F variant, the lower sensitivity to tebuconazole of I381V variants, increased resistance to prochloraz of V136A variants together with sensitivity to triadimenol and tebuconazole; and finally, resistance to tebuconazole and decreased sensitivity to epoxiconazole of the A379G I381V ΔY459/G460 combination coupled to sensitivity to prochloraz.

We provide detailed descriptions for how different combinations of mutations affect binding of four different azole fungicides based on molecular models of CYP51. All these findings support the idea that the different binding specificities of different azoles to particular CYP51 variants has contributed to observed amount of polymorphism in the CYP51 gene, as a result of the mosaic of different fungicides that have been applied in wheat fields of Europe.

The structure – function relationships underpinning the observation that resistance of new combinations of mutations to new azole fungicides may increase sensitivity to older azole fungicides might inform effective rotation of fungicides or could be exploited to create new fungicide mixtures.

The measurement of the volume of the haem cavity was a useful metric for assessment of the scope for general resistance to this group of compounds by virtue of their accommodation without the potential for key binding interactions. It appears that this combination of methods provides the basis to a reliable *in silico* predictive approach for assessing the probability of particular variants exhibiting resistance to particular azole fungicides that would be of value in predicting and managing the use of fungicides in the control of this important wheat pathogen. This approach to understanding binding of azoles can be used as a screen to identify new fungicidal compounds and also to predict which azole classes will be affected by any particular combination of CYP51 mutations.

In terms of studying protein evolution more generally, our analyses have provided much insight into the role of specific amino acids in binding azoles in *M. graminicola*, and how conformational changes brought about by the alteration of particular residues, often proximal to the ligand, can result in modified binding capacity of other key residues. In doing so, we have established a promising *in silico* protocol for the assessment of alterations and their effects in proteins where evolutionary stress is introduced by the extensive application of a closely related class of agents targeting a specific protein binding domain.

## Supporting Information

Table S1 **Proximities of residues to bound azoles in modelled** ***M. graminicola*** **CYP51 variants.** Residues in positions that are incorporated in the alterations are underlined.
(DOCX)

Table S2 **Frequency of residues within interaction range of bound azoles.** Residues within 4.5 A of docked azoles, how frequently they appear, their predicted location within CYP51 (according to alignment with *Mtb*). Residues observed as point mutations in CYP51 are shown in bold.
(DOCX)

Text S1 **Residues lining the binding pocket of** ***Mycosphaerella graminicola*** **CYP51 wild type protein.**
(DOCX)
