# Supplementary material for: Molecular Modelling of the Emergence of Azole Resistance in Mycosphaerella graminicola
Source: PLoS One. 2011 Jun 27;6(6):e20973. doi: 10.1371/journal.pone.0020973 (PMC3124474; doi:10.1371/journal.pone.0020973)
Supplement: Table S1 — Proximities of residues to bound azoles in modelled M. graminicola CYP51 variants. Residues in positions that are incorporated in the alterations are underlined. (DOCX) [file pone.0020973.s001.docx]

| Supplementary Table 1: Proximities of residues to bound azoles in modelled *M. graminicola* CYP51 variants | | | | | | | | | | | | | | | | | | | |
| --- | --- | --- | --- | --- | --- | --- | --- | --- | --- | --- | --- | --- | --- | --- | --- | --- | --- | --- | --- |
|  | **Epoxiconazole** | | | | **Prochloraz** | | | | | **Tebuconazole** | | | | | **Triadimenol** | | | | |
| **Variant** | <3.0Å | 3.0-3.5Å | 3.5-4.5Å |  | | <3.0Å | 3.0-3.5Å | 3.5-4.5Å |  | | <3.0Å | 3.0-3.5Å | 3.5-4.5Å |  | | <3.0Å | 3.0-3.5Å | 3.5-4.5Å |  |
| wt | **Y459**A307 M310 A311 E457 D458 | Y123 **V136** **G460** | L309 **Y461** |  | | M310 **I381** D458 **Y459**  **G460** | A311 | Y123 L309 P380 E457 **Y461** |  | | D458Y123 M310 A311 E457 **Y459** | L309 **G460** | **V136** G312 Q313 **Y461** |  | | **Y459**Y123 D458 **G460** | **V136** E457 | D134  **Y137** A307 A311 P380 **I381** **Y461** |  |
| L50S | M310 E457 D458 **Y459** | P125 A311 **G460** | Y123 **Y137** **I381** **Y461** |  | | M310 **I381** D458 **Y459** | **G460** | Y123 A311P380 E457 **Y461** |  | | D458M310  E457 **Y459** | P125 **G460** | Y123 S124 **Y137** L309 A311 Q313 **I381** **Y461** |  | | **I381** E457 D458 **Y459** | **G460** | Y123 **Y137** A311 **Y461** |  |
| Y137F | **F137**M310 A311 | A307 | Y123 L309 H314 **Y461** |  | | M310H314 **I381** | A311 | **F137** L309 Q313 S315 **G460** **Y461** |  | | Y123 **F137** M310 A311 | L309 | G312 Q313 H314 **Y461** |  | | **F137 I381** | Y123 | A311 H314 **Y459** **G460** **Y461** |  |
| Y459D | I122 Y123 M310 A311 L463 V464 | E121 H314 | A307 G462 |  | | L463M310 H314 **I381**  V464 | Y226 | E121 I122 Y123 A311 S315 P380 K466 |  | | V464E121 M310 L463 | I122 A311 H314 | Y123 Y226 L240 L309 G312 Q313 **I381**  G462 |  | | E121 **I381** L463 V464 |  | I122 D134 L240 A311 H314 S383 **Y461** G462 S520 |  |
| G460D | **Y137**M310 A311 D458 **Y459** **D460** | A307 | L309 E457 |  | | **Y137** M310 **I381** D458 **Y459** **D460** | A311 H314 | L309 **Y461** L463 |  | | **Y137**  **D460**L309 M310 A311 D458 **Y459** | G312 Q313 | E457 |  | | **Y137**  **D460 I381** D458 **Y459** | D134 H314 D458 **Y461** | A311 |  |
| L50S Y461H | M310Y123 **Y137** Y253 A311 | F131 A307 **Y459** | L309 **G460** **H461** |  | | M310Y123 **I381**  **Y459** | **G460** | Y253 L309 A311 H314 S315 **H461** L463 C482 |  | | Y123M310 A311 | **Y137** L309 Q313 **Y459** | S124 G312 D458 **G460** **H461** |  | | Y123  **Y137** D458 **Y459** | **I381** | F131 A307 M310 A311 H314 **G460** |  |
| L50S Y461S | I122  Y123 M310 A311 D458 | A307 H314 **Y459** | E121 S124 L309 **G460** |  | | Y123 M310 H314 **I381**  D458  **Y459** | **G460** | I122 L309 A311 P380 |  | | Y123  I122 M310 D458 **Y459** | L309 A311 H314 E457 | E121 S124 G312 Q313 **G460** |  | | I122D458 **Y459** | **G460** | E121 Y123 D134 **V136** A311 H314 **I381** E457 |  |
| L50S I381V Y461H | **Y137**  Y123 M310 A311 H314 D458 **Y459** |  | F131 D134 **V136** A307 L309 S315 **H461** |  | | D458  **Y137** M310 H314 **V381** **Y459** | Y123 **G460** | L309 A311 S315 P380 **H461** L463 |  | | Y123  **Y137** M310 H314 D458 **Y459** | A311 Q313 | **V136** L309  G312 E457  **H461** |  | | D458Y123 **Y137** **V381** E457 **Y459** | D134 | F131 **V136** A311 H314 S315 **G460** **H461** |  |
| L50S V136A Y461H | **Y137** D458Y123 M310 A311 **Y459** **G460** **H461** |  | I122 S124 P125 L126 A307 H314 S315 |  | | **Y459**Y123 M310 H314  **G460** **H461** | P125 **I381** D458 | I122 **Y137** A311 S315 P380 |  | | Y123 M310 D458 **Y459** **G460** **H461** | S124 P125 **Y137** A311 Q313 | I122 L126 L309 G312 H314 E457 |  | | **Y459**Y123 S124 P125 **Y137** D458 **G460** **H461** |  | I122 A311 H314 S315 P380 **I381** |  |
| L50S S188N N513K | **Y137**  M310 | A307 A311 **I381** | **V136** Y253 L309 K456 E457 K466 |  | | **I381**M310 | **Y137** Y226 | A311 P380 S465 K466 |  | | **Y137**M310 E457 | A311 **I381** | **V136** Y226 L309 G312 Q313 K466 |  | | **Y137** **I381** | **V136** E457 | D134 A311 D458 K466 |  |
| L50S S188N ΔY459/G460 N513K | Y123 M310 A311 | H314 | I122 A307 L309 |  | | M310 H314 **I381** | Y226 | E121 Y123 A311 |  | | I122 Y123 M310 | A311 H314 | Y226 L309 G312 Q313 |  | | I122 M310 | Y123 **I381** | E121 M310 A311 H314 |  |
| L50S S188N I381V ΔY459/G460 N513K | Y123L126 M310 A311 | A307 | L309 H314 |  | | Y123 M310 H314 **V381** | A311 | L309 |  | | Y123 M310 A311 | Q313 | I122 L126 L309 G312 H314 |  | | Y123 | H314 **V381** | I122 A311 |  |
| L50S V136A S188N ΔY459/G460 N513K | M239 M310 A311 | K148 F238 W242 A243  P244 | I236 L240 A307 L309  H314 |  | | M239  M310 A311 **I381** | H314 | K148 I236  L240 L309 S315 P380 |  | | K148 M239 L240 M310 | A311 | I236 F238 P241 L309 G312 Q313 H314 |  | | M239 | K148 L240 | K133 W242 W243 M310 A311  H314 **I381** S383 |  |
| L50S S188N A379G I381V ΔY459/G460 N513K | Y123 A307 M310 A311 |  | D134 **V136** **Y137** L309 H314 |  | | M310H314 **V381** |  | L309 A311 |  | | M310 A311 | L309 Q313 | **V136** **Y137** G312 H314 |  | | D134 **Y137** | **V136** H314 | A307 A311 **V381** |  |

Residues in positions that are incorporated in the alterations are underlined.
