# Supplementary material for: Molecular Modelling of the Emergence of Azole Resistance in Mycosphaerella graminicola
Source: PLoS One. 2011 Jun 27;6(6):e20973. doi: 10.1371/journal.pone.0020973 (PMC3124474; doi:10.1371/journal.pone.0020973)
Supplement: Table S2 — Frequency of residues within interaction range of bound azoles. Residues within 4.5 A of docked azoles, how frequently they appear, their predicted location within CYP51 (according to alignment with Mtb). Residues observed as point mutations in CYP51 are shown in bold. (DOCX) [file pone.0020973.s002.docx]

Supplementary Table 2. Frequency of residues being within interaction range of bound azoles

|  |  |  | frequency (of 56) |
| --- | --- | --- | --- |
| E | 121 | close to SRS1 | 9 |
| I | 122 | adjacent to SRS1 | 17 |
| Y | 123 | SRS1 | 39 |
| S | 124 | SRS1 | 7 |
| P | 125 | SRS1 B' helix | 6 |
| L | 126 | SRS1 B' helix | 4 |
| F | 131 | SRS1 B' helix | 4 |
| K | 133 | SRS1 | 1 |
| **D** | **134** | SRS1 | 9 |
| **V** | **136 /A136** |  | 15 / 0 |
| **Y** | **137 /F137** |  | 27 / 4 |
| K | 148 |  | 4 |
| Y | 226 | SRS2 F helix | 6 |
| I | 236 |  | 3 |
| F | 238 |  | 2 |
| M | 239 |  | 4 |
| L | 240 |  | 6 |
| P | 241 |  | 1 |
| W | 242 |  | 2 |
| A | 243 |  | 1 |
| P | 244 |  | 1 |
| Y | 253 | SRS3 G helix | 3 |
| A | 307 | SRS4 I helix | 16 |
| L | 309 | SRS4 I helix | 34 |
| M | 310 | SRS4 I helix | 46 |
| **A** | **311** | SRS4 I helix | 56 |
| **G** | **312** | SRS4 I helix | 13 |
| Q | 313 | SRS4 I helix | 15 |
| H | 314 | SRS4 I helix | 40 |
| S | 315 | SRS4 I helix | 10 |
| P | 380 | SRS5 | 10 |
| **I** | **381/V381** |  | 30/6 |
| S | 383 | SRS5 | 2 |
| K | 456 | fungi specific region | 1 |
| E | 457 | fungi specific region | 18 |
| D | 458 | fungi specific region | 28 |
| **Y** | **459/C459/D459** | fungi specific region | 34 / 0 / 0 |
| **G** | **460/D460** | fungi specific region | 29 / 4 |
| **Y** | **461/H461/S461** | fungi specific region | 19 / 11 / 0 |
| G | 462 | fungi specific region | 3 |
| L | 463 | fungi specific region | 7 |
| V | 464 | fungi specific region | 4 |
| S | 465 | fungi specific region | 1 |
| K | 466 | fungi specific region | 5 |
| C | 482 | haem binding cysteine | 1 |
| S | 520 | SRS6 | 1 |

Residues within 4.5 A of docked azoles, how frequently they appear, their predicted location within CYP51 (according to alignment with *Mtb)*. Residues observed as point mutations in CYP51 are shown in bold.
