# Supplementary material for: Molecular Modelling of the Emergence of Azole Resistance in Mycosphaerella graminicola
Source: PLoS One. 2011 Jun 27;6(6):e20973. doi: 10.1371/journal.pone.0020973 (PMC3124474; doi:10.1371/journal.pone.0020973)
Supplement: Text S1 — Residues lining the binding pocket of Mycosphaerella graminicola CYP51 wild type protein. (DOCX) [file pone.0020973.s003.docx]

Supplementary information

Residues lining the binding pocket of *Mycosphaerella graminicola* CYP51 wild type protein

The residues (and sections) lining the pocket of the wild type protein are I109, N111, G112, K113, L114, V117, N118, A119, E121, I122, Y123 (section 109-123), V135, V136 (section 135-136, adjacent to Y137), L144, M145, K148, K149, F150, V151, K152, Y153, G154, L155 (section 144-155), A159, Y163, L197, T201, I202, A205, S206, S208, L209, Q210 (section 201-210), F218, Y228, Y262, I266, E278, E279, N284, M286, Q287, I301, A302, M304, M305, I306, A307, L308, L309, M310, A311, G312, Q313, S315, S316, T319, E320 (section 302-320), V371, K372, L375, I381, S383, I384, L385, R386 (section 381-386), E457, Y459, G460, Y461, G462 (section 457-462), Y472, F475, R479, H480, R481, C482, I483, G484, E485, F487, A488, Y489, Q491, L492 (section 479-492), I495; several of which are, or are close to, residues subject to mutation
